# Downregulation of lncRNA CCAT1 enhances 5-fluorouracil sensitivity in human colon cancer cells

**DOI:** 10.1186/s12860-019-0188-1

**Published:** 2019-04-23

**Authors:** Chun Yang, Yong Pan, Shao Ping Deng

**Affiliations:** Department of Gastrointestinal Surgery, Sichuan Academy of Medical Sciences & Sichuan Provincial People’s Hospital, School of Medicine, University of Electronic Science and Technology, No.32 Western Section 2 Yihuan Road, Chengdu, 610072 Sichuan China

**Keywords:** Colon cancer, lncRNA CCAT1, 5-fluorouracil, Apoptosis

## Abstract

**Background:**

The purpose of this study was to determine the aberrant expression of the long noncoding RNA (lncRNA) colon cancer-associated transcript 1 (CCAT1) in 5-fluorouracil-resistant colonic neoplasm cells and to elucidate its effects on the 5-fluorouracil sensitivity of human colonic neoplasm cells. The aberrant expression of lncRNAs in normal tissues and colonic neoplasm tissues was detected by microarray assay. qRT-PCR analysis was performed to assess CCAT1 expression levels in colonic neoplasm cell lines and corresponding normal tissues. After constructing the 5-FU-resistant cell lines and validating the resistance by measuring the IC_50_ value, the CCAT1 expression levels in parental and artificially resistant cell lines were determined by qRT-PCR. Transfection was used to modulate the expression of CCAT1. Cell proliferation and apoptosis were then detected by CCK-8 and flow cytometry, respectively.

**Results:**

CCAT1 in colon cancer tissues was higher than that in noncancer tissues, and the levels of CCAT1 in HCT 116, SW1417, HT-29, and KM12 cell lines were higher than those in the human normal colon epithelial NCM460 cell line. Moreover, the expression levels of CCAT1 were high in HCT 116/5-FU and HT-29/5-FU cell lines, whose apoptosis rates induced by 5-FU were lower than those in corresponding parental cells. The results of qRT-PCR and CCK-8 assay showed that enhancement of lncRNA CCAT1 expression levels in HCT 116 and HT-29 cell lines increased their IC_50_ of 5-FU and decreased their apoptosis rates. Meanwhile, siRNA-CCAT1 effectively inhibited the expression of CCAT1 and enhanced the 5-FU-sensitivity of HCT 116/5-FU and HT-29/5-FU, in which apoptosis rates were increased at the same time.

**Conclusions:**

Downregulation of CCAT1 effectively reversed the resistance of HCT 116/5-FU and HT-29/5-FU cells to 5-FU chemotherapeutic, opening a new avenue in colon cancer therapy.

## Background

Colon cancer is a common malignant tumor of the digestive tract that occurs predominantly at the junction of the rectum and the sigmoid colon, with the highest incidence in the 40-to-50-year-old age group [1]. Colon cancer accounts for one-third of all malignant tumors in the world and ranks fourth in mortality. It is mainly divided into adenocarcinoma, mucinous adenocarcinoma, and undifferentiated carcinoma. The general shape of tumors is polypoid or ulcers [2]. Patients with chronic colitis, colon polyps, and obese men are predominantly susceptible [3]. Although nonspecific cytotoxicity narrows its clinical therapeutic index, leading to small differences between therapeutic and toxic doses, treatment resistance to 5-FU often occurs and results in poor prognosis for patients [4]. Thus, further understanding of the molecular basis that accounts for the chemotherapeutic resistance is still needed.

Long-chain noncoding RNAs (lncRNAs) are a class of RNA molecules with transcripts over 200 nt in length. Although they do not encode proteins, lncRNAs are expressed on multiple levels (epigenetic regulation, regulation of transcription and posttranscriptional, etc.) in forms of RNA to regulate the expression of related genes [1]. Thus far, relationships between occurrences of many tumors and lncRNAs have been elucidated. For example, abnormal expression of lncRNAs has been observed in many solid tumors, such as colon cancer, non-small cell lung cancer and ovarian cancer and breast cancer [5]. Until now, it has been found that more than 7000 lncRNAs are functional, and some lncRNAs can be used as indicators of tumor diagnosis and monitoring progress and can provide points for tumor treatment [6].

CCAT1, located on human chromosome 8q24.21, is described as a “hot spot,” which leads to genetic mutations in colon cancer [7]. Studies of human tissues found that the smallest CCAT1 is expressed poorly in normal liver tissues and small intestine tissues, and many other human tissues have not found any expression of CCAT1 [7]. Compared with that in normal tissues, CCAT1 was demonstrated to be overexpressed in colonic neoplasm tissues, which promoted the proliferation and the invasion of colonic neoplasm cells. Clinically, CCTA1 is closely related to the lymph node metastasis, clinical stage and prognosis of patients [8]. Sun et al. found that CCAT1 is a potential biomarker of colonic neoplasms, which indicated that CCAT1 could be used to predict the colorectal cancer prognosis [9]. Nissan et al. reported that CCAT1 is a highly specific and readily detectable marker for CRC and tumor-associated tissues [10]. However, little is known about the expression levels of CCAT1 in colonic carcinoma or whether CCAT1 is involved in the progression of chemoresistance.

Traditional chemotherapy drugs and new biological target therapy are important treatment methods for colonic cancer. In the classic chemotherapy regimen, the effective rate of 5-fluorouracil (5-FU) monotherapy for advanced colon cancer patients is only 10–16% [11]. Combined with other drugs, such as irinotecan and oxaliplatin, the effective rate of 5-FU is less than 50% [12]. Currently, the decline in chemosensitivity is the main reason for the poor response to chemotherapy in colonic neoplasms [13].

In this study, the effect of CCAT1 on the chemosensitivity of colonic neoplasm cells to 5-FU was determined. We found that downregulation of CCAT1 effectively enhanced the chemosensitivity of 5-FU-resistant colon cancer cells, providing a new avenue for colon cancer therapy.

## Results

### CCAT1 is upregulated in human colonic neoplasm tissues

**Fig. 1:**
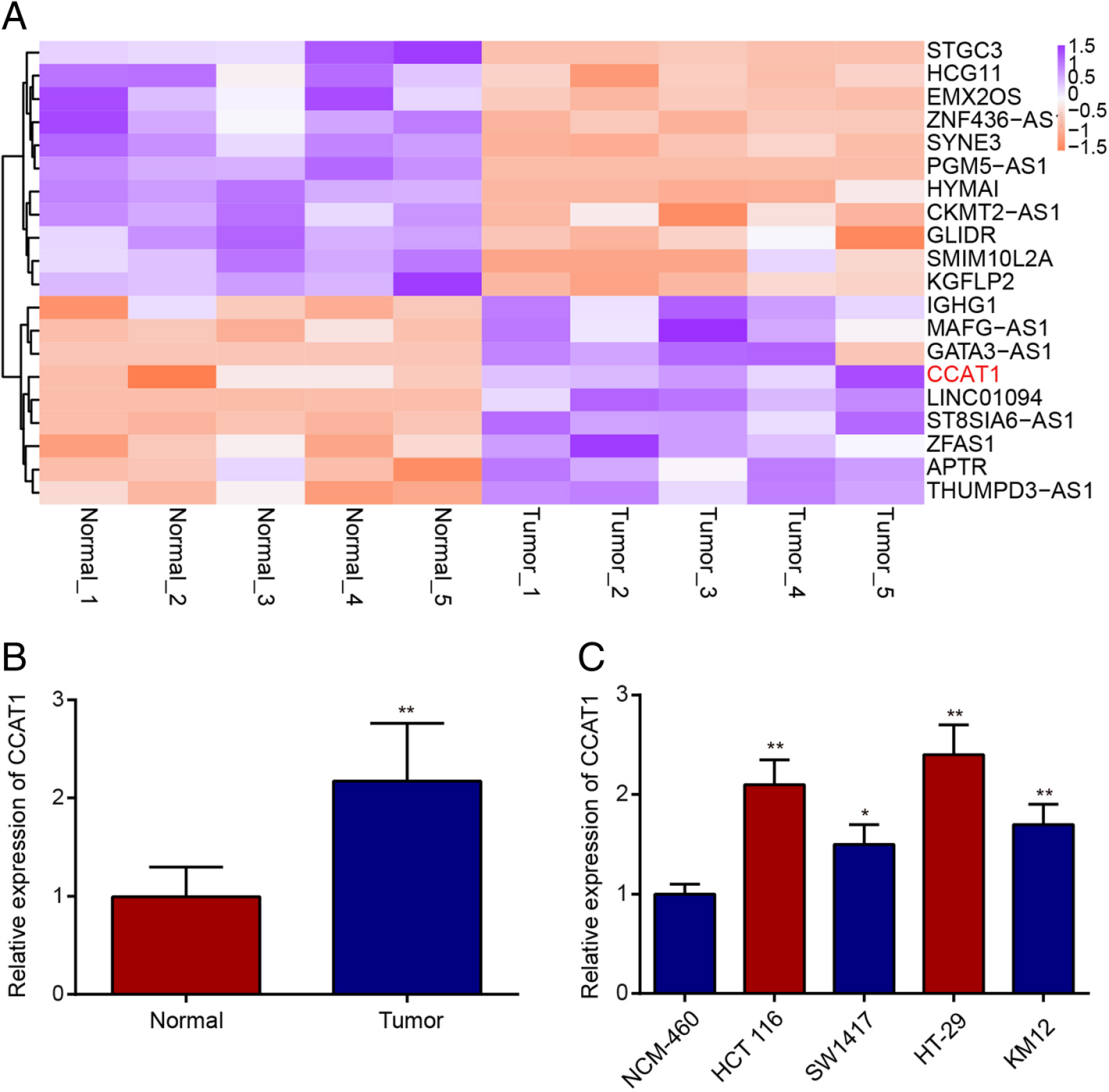
lncRNA CCAT1 was overexpressed in colon cancer. **a**. Heat map analysis of the lncRNA expression in cancerous and adjacent tissues was created using a method of hierarchical clustering by GeneSpring GX, version 7.3. Red: greatest, Green: lowest. **b**. We validated the differential expression of lncRNA CCAT1 in paired colon cancer and adjacent samples using RT-PCR. ^**^P < 0.01 compared with the normal group. **c.** The expression of lncRNA CCAT1 in different colon cancer cell lines and NCM460 cells were detected by qRT-PCR. Data are presented as the mean ± SD. ^*^P < 0.05, ^**^P < 0.01 compared with the NCM460 group

In our study, the differentially expressed lncRNAs in 67 pairs of colon cancer tissues and pair-matched adjacent normal tissues were screened using microarray analysis. As presented in Fig. 1a, CCAT1 expression level was upregulated. To validate the microarray analysis results, the expression of CCAT1 was also examined by qRT-PCR. Compared with those in matched noncancerous tissue (Fig. 1b), the levels of CCAT1 were increased in cancerous tissues. Next, we detected the expression of CCAT1 in some representative colon cancer cell lines (HCT 116, SW1417, HT-29, and KM12) and human normal colonic epithelial cell line (NCM460). As Fig. 1c shows, the expression of CCAT1 exhibited the greatest upregulation in both HCT 116 and HT-29 colon cancer cell lines compared to the normal cell line.

### CCAT1 is upregulated in 5-FU-resistant colonic neoplasm cell lines

**Fig. 2:**
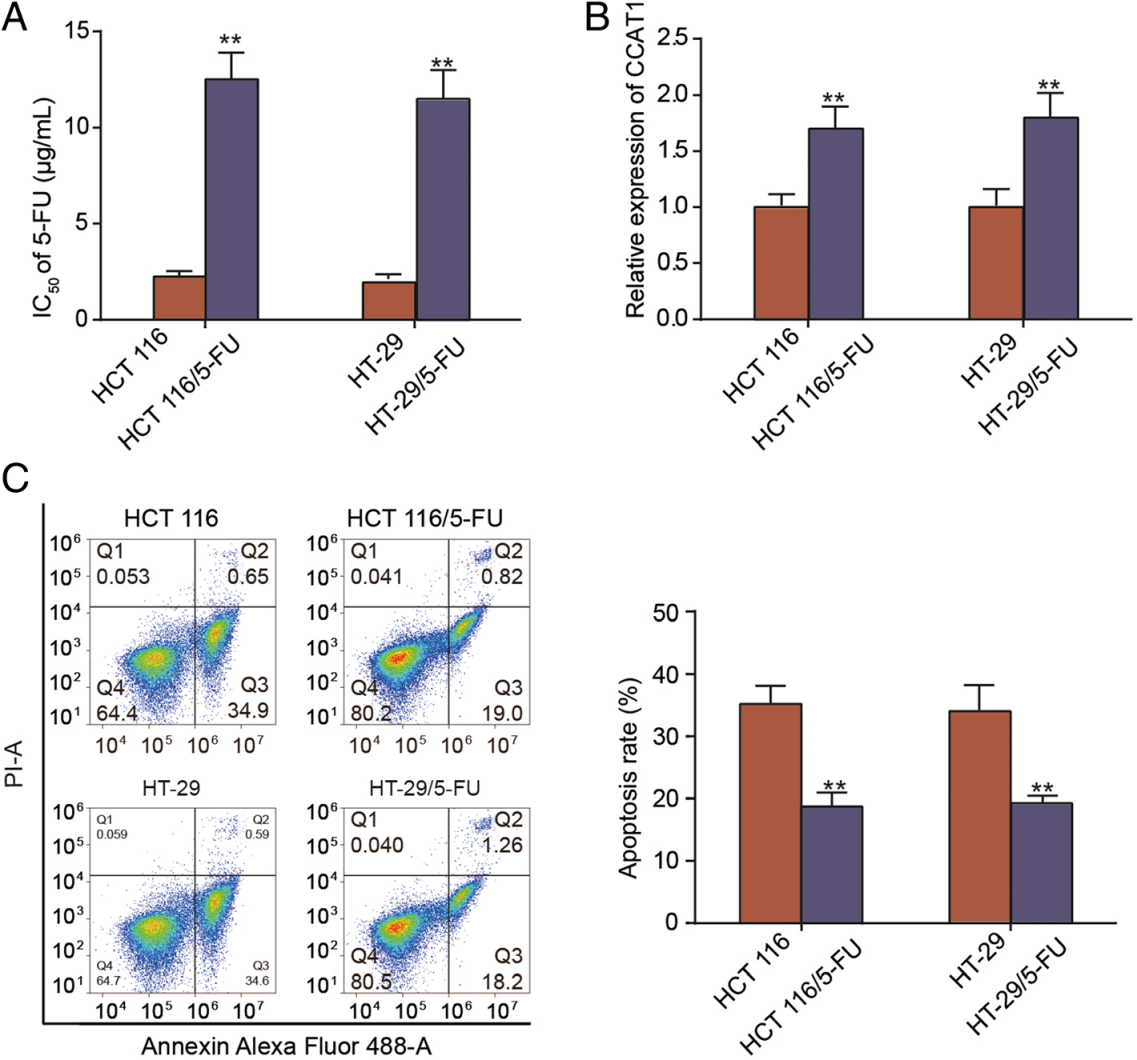
lncRNA CCAT1 was overexpressed in 5-FU resistant colon cancer cells. **a**. CCK-8 essay was performed to examine the IC50 value for HCT 116, HT-29, HCT 116/5-FU and HT-29/5-FU cells after 5-FU treatment. **b.** The expression of CCAT1 in HCT 116/5-FU, HT-29/5-FU cell lines were determined by qRT-PCR. **c.** Flow cytometry analysis was used to detected the apoptosis rate of HCT 116, HT-29, HCT 116/5-FU and HT-29/5-FU cells after 5-FU treatment. Error bars represent the mean ± SD. ^**^P < 0.01 compared with the HCT 116 or HT-29 group

Chemoresistance is still one of the major barriers in the clinical treatment of colonic neoplasms. Moreover, CCAT1 was found to be involved in the chemoresistance in NSCLCs [14], lung adenocarcinoma cells [15] and nasopharyngeal cancer cells [16]. To determine whether CCAT1 is involved in the chemoresistance to colonic neoplasms, first two 5-FU-resistant cell lines HCT 116/5-FU and HT-29/5-FU were established based on previous research [17]. We used the CCK-8 assay to confirm the relative chemosensitivity of these 5-FU-resistant cell lines (Fig. 2a). The IC_50_ values for 5-FU in HCT 116/5-FU and HT-29/5-FU cell lines were 12.5 ± 1.4 μg/ml and 11.5 ± 1.5 μg/ml, respectively. And the respective corresponding values for their paired cells HCT 116 and HT-29 cells, were 2.25 ± 0.3 μg/ml and 1.95 ± 0.25 μg/ml, respectively, which were importantly lower than those of HCT 116/5-FU and HT-29/5-FU cells. In addition, we used flow cytometry analyses to evaluate the degree of apoptosis in these drug-resistant cell lines. We found that the apoptosis rates in HCT 116/5-FU and HT-29/5-FU cell lines were lower than those of parental cell lines (Fig. 2c).

Subsequently, the expression of CCAT1 in corresponding parental cell lines (HCT 116 and HT-29) and two 5-FU resistant colon cancer cell lines (HCT 116/5-FU and HT-29/5-FU) were examined. As showed in Fig. 2b, expression of CCAT1 was upregulated in 5-FU-resistant colonic neoplasm cell lines compared with parental cell lines. In general, these abovementioned data indicated that the expression of CCAT1 in 5-FU-resistant colonic neoplasm cell lines was greater than the parental cell lines, meaning that CCAT1 was active in 5-FU resistant cells, and decreased chemosensitivity of colonic neoplasm cell in vitro, and reduced apoptosis at the same time.

### Expression of CCAT1 accounts for 5-FU sensitivity of HCT 116 (or HT-29) cell lines

**Fig. 3:**
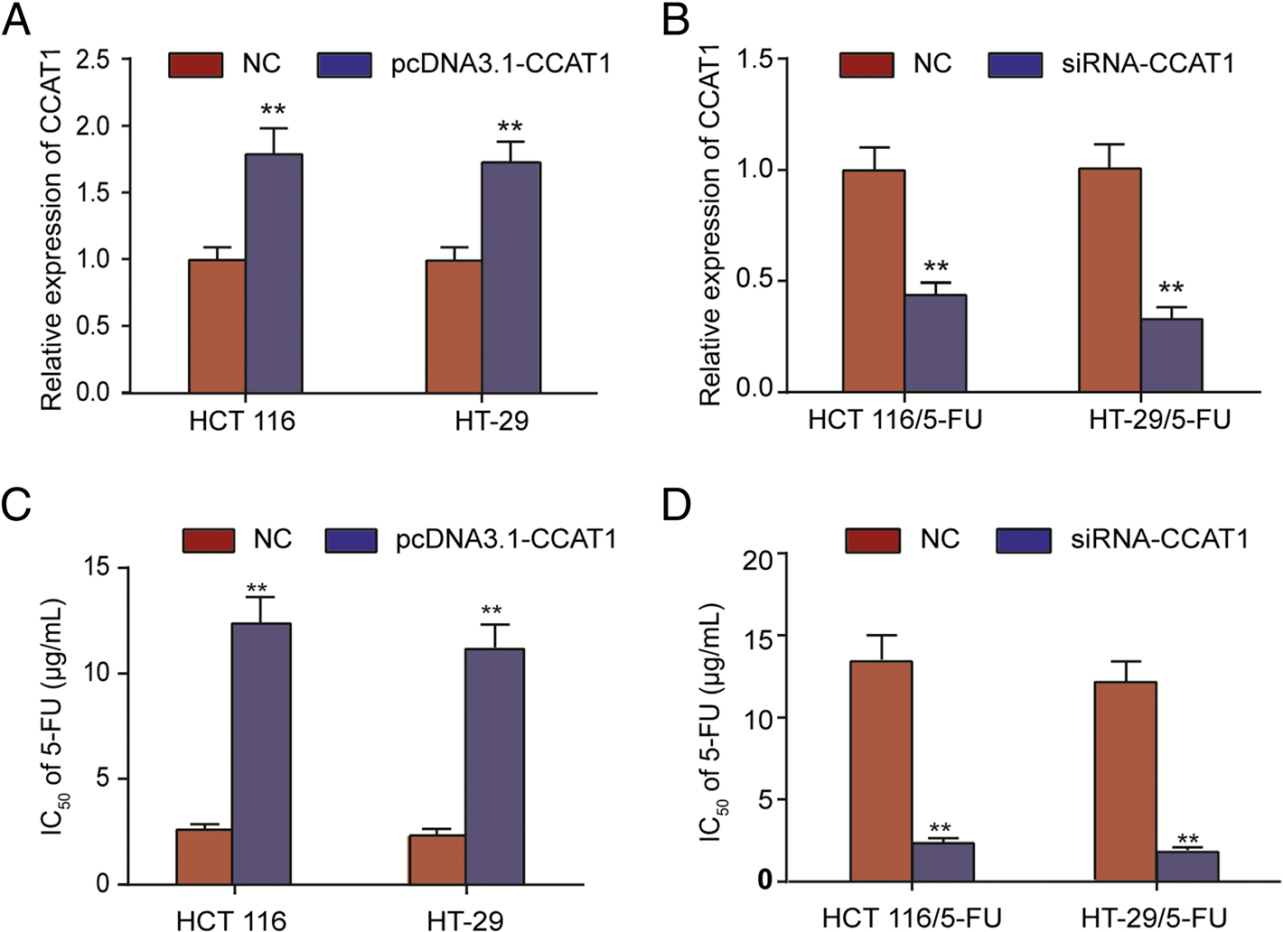
Role of CCAT1 in chemosensitivity of parental or 5-FU-resistant colon cancer cell lines. **a**, **b**. qRT-PCR assay was performed to examine the expression of CCAT1 after the transfection of HCT 116 or HT-29 cells with pcDNA3.1-CCAT1 and of HCT 116/5-FU or HT-29/5-FU cells with siRNA-CCAT1. **c, d.** IC50 values for 5-FU in HCT 116 or HT-29 cells transfected with pcDNA3.1-CCAT1 and HCT 116/5-FU or HT-29/5-FU cells transfected with siRNA-CCAT1. ^**^P < 0.01 compared with respective NC group

To investigate biological functions of CCAT1 in colonic neoplasm cells chemoresistance against 5-FU, HCT 116 (or HT-29) and HCT 116/5-FU (or HT-29/5-FU) cells were stably transfected with the CCAT1 expression vector pcDNA3.1-CCAT1 or the specific siRNA of CCAT1, while the empty vector or control siRNA, respectively, were used as a negative control (NC). The satisfactory efficiency of transfection was obtained at 48 h after transfection. We observed that pcDNA3.1-CCAT1 accelerated the expression level of CCAT1 in colonic neoplasm cell lines HCT 116 and HT-29, while siRNA-CCAT1 markedly repressed the CCAT1 level in 5-FU-resistant cell lines (Fig. 3a, b).

We then detected the change of IC_50_ values of 5-FU in conditions of overexpression and downregulation of CCAT1. The IC_50_ value of 5-FU in HCT 116 or HT-29 cells increased with the upregulation of CCAT1 (Fig. 3c) compared with that of HCT116/control or HT-29/control cells, respectively. Furthermore, the IC_50_ value of HCT 116/5-FU or HT-29/5-FU transfected with si-CCAT1 was reduced compared with that of HCT 116/5-FU or HT-29/5-FU cells, respectively, transfected with the negative control, indicating that si-CCAT1 might act as a promoter of 5-FU sensitivity (Fig. 3d). In summary, these results indicated that CCAT1 inhibited 5-FU sensitivity of HCT116 and HT-29 cell lines.

### Influences of CCAT1 expression on apoptosis rates

**Fig. 4:**
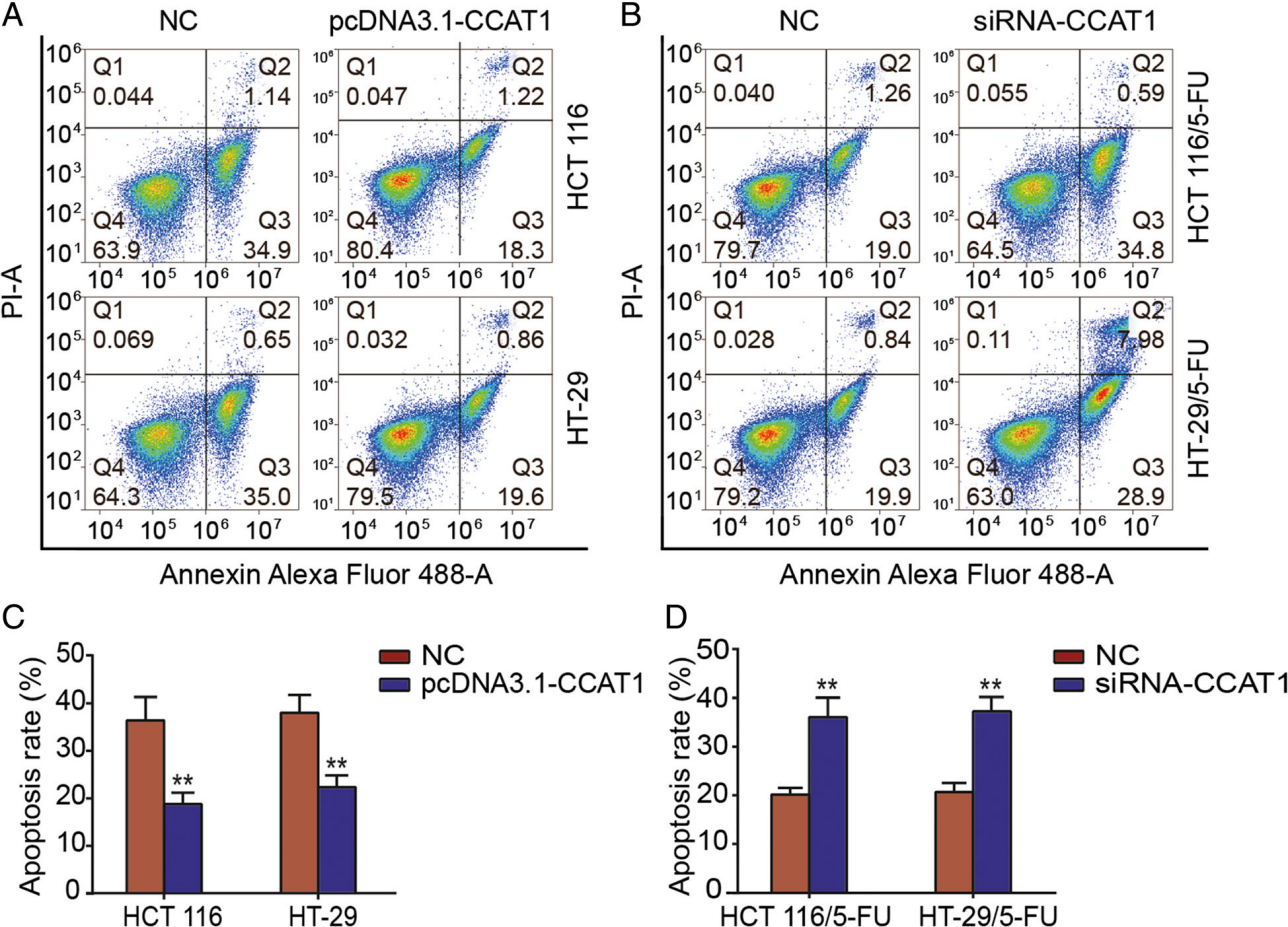
Knockdown of CCAT1 reverses drug resistance in 5-FU-resistant colon cancer cell lines. **a**, **c**. Apoptosis of HCT 116 or HT-29 cells transfected with pcDNA3.1-CCAT1 were assessed using flow cytometry. **b, d.** Apoptosis of HCT 116/5-FU or HT-29/5-FU cells transfected with siRNA-CCAT1 were assessed using flow cytometry. Error bars represent the mean ± SD. ^**^P < 0.01 compared with the respective NC group

In addition, to further investigate the potential associations between the expression level of CCAT1 and 5-FU resistance, flow cytometry was used to assess the apoptosis rate. Enhanced expression of CCAT1 resulted in a decrease in apoptosis in HCT 116 or HT-29 cells treated with 5-FU (2 μg/mL) compared with negative controls (Fig. 4a, c). Similarly, downregulation of CCAT1 resulted in a significant increase in apoptosis rates of HCT 116/5-FU or HT-29/5-FU cell lines compared to the NC groups as 5-FU cell lines were exposed to 5-FU (2 μg/mL) (Fig. 4b, d). In conclusion, the knockdown of CCAT1 was able to significantly reverse the drug resistance of 5-FU-resistant colonic neoplasm cell lines by inducing apoptosis.

### Mechanism of CCAT1 affects the sensitivity of cells to 5-FU

**Fig. 5:**
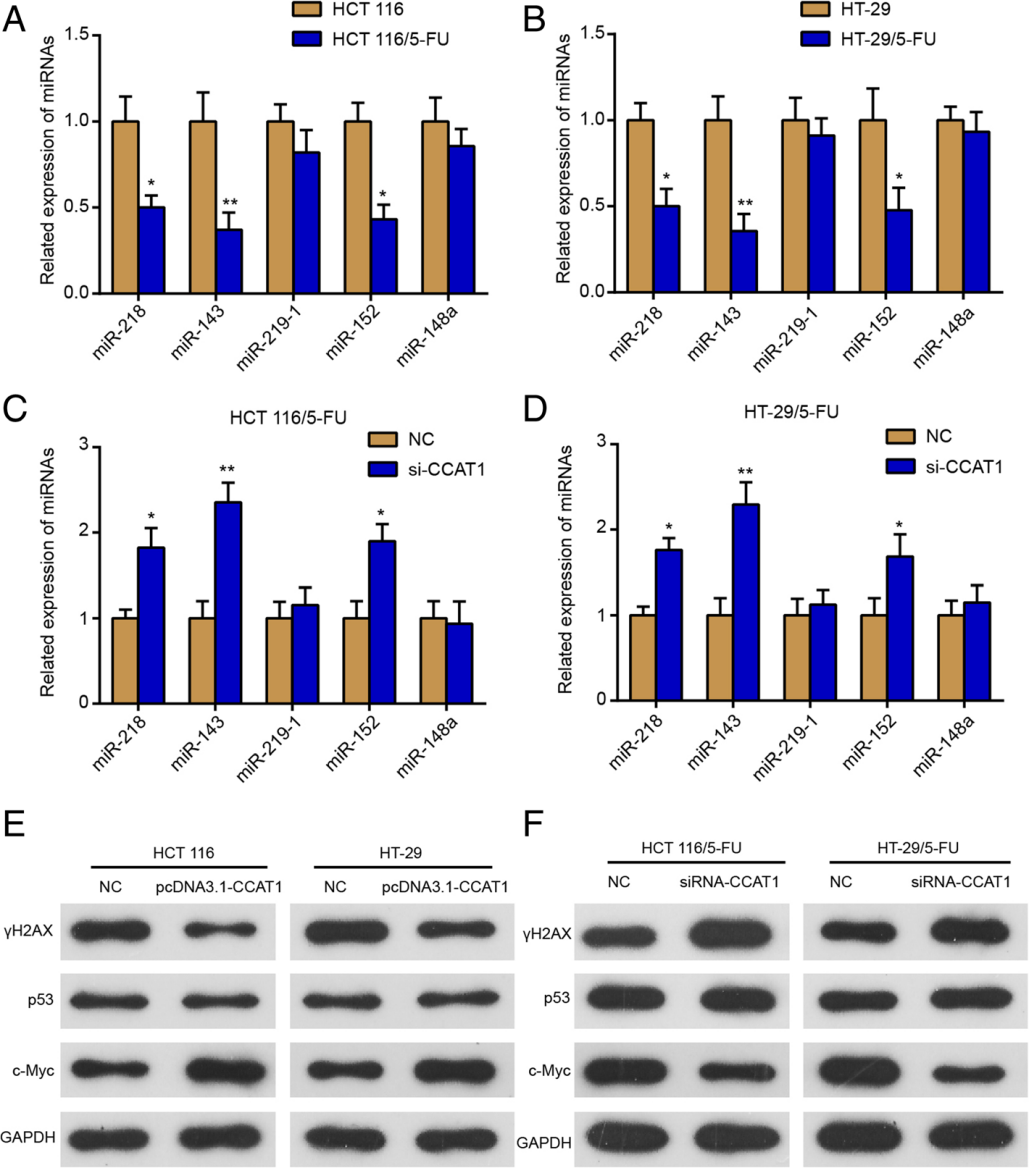
Mechanism of CCAT1 on the sensitivity of cells to 5-FU. **a.** The relative expression of miRNAs in HCT 116 and HCT 116/5-FU cells was assessed using qRT-PCR. **b.** The relative expression miRNAs in HT-29 and HT-29/5-FU cells was determined by qRT-PCR. **c.** The relative expression of miRNAs in HCT 116/5-FU cells was determined using qRT-PCR after siRNA-CCAT1 transfection. **d.** The relative expression of miRNAs in HT-29/5-FU cells was examined by qRT-PCR after siRNA-CCAT1 transfection. **e.** The protein expressions of γ-H2AX, p53 and c-Myc in HCT 116 and HT-29 cell lines were determined by Western blot after pcDNA3.1-CCAT1 transfection. **F.** HCT 116/5-FU and HT-29/5-FU cells were transfected with siRNA-CCAT1, then the protein levels of γ-H2AX, p53 and c-Myc were evaluated by Western blot. ^*^P < 0.05, ^**^P < 0.05, compared with HCT 116, HT-29 or NC group. All data were means ± SD

To figure out the mechanism of CCAT1 on the sensitivity of cells to 5-FU, we detected the expression of some miRNAs, which were confirmed to be targets of CCAT1 in other cancers [18–20]. As shown in Fig. 5a and b, the expression of miR-218, miR-143 and miR-152 was significantly decreased in HCT 116/5-FU and HT-29/5-FU cells compared with HCT 116 or HT-29 cells, respectively, while there was no significant difference in the expression of miR-219-1 and miR-148a. Knockdown of CCAT1 increased the expression of miR-218, miR-143 and miR-152, but not miR-219-1 or miR-148a, in HCT 116/5-FU (Fig. 5c) and HT-29/5-FU (Fig. 5d) cells. All the abovementioned results indicated that CCAT1 may regulate the expression of miR-218, miR-143 and miR-152 as miRNA sponges to have an effect on 5-FU sensibility of human colon cancer cells. In addition, we detected the protein expression of γ-H2AX, p53 and c-Myc in HCT 116 and HT-29 cells treated with 5-FU (2 μg/mL) (Fig. 5e) and in HCT 116/5-FU and HT-29/5-FU cells treated with 5-FU (2 μg/mL) (Fig. 5f) after transfection. The protein expression of γ-H2AX, a DNA damage marker, was negatively correlated with CCAT1 expression, but there was no significant difference in the expression of p53 after transfection, which indicated that CCAT1 promoted DNA damage to promote 5-FU sensibility in human colon cancer cells. In addition, we also found the protein expression of c-Myc was positively correlated with CCAT1 expression, which correlated with the mechanism of CCAT1-modulated 5-FU sensibility in human colon cancer cells.

### Tumor growth and the expression of CCAT1 in vivo

**Fig. 6:**
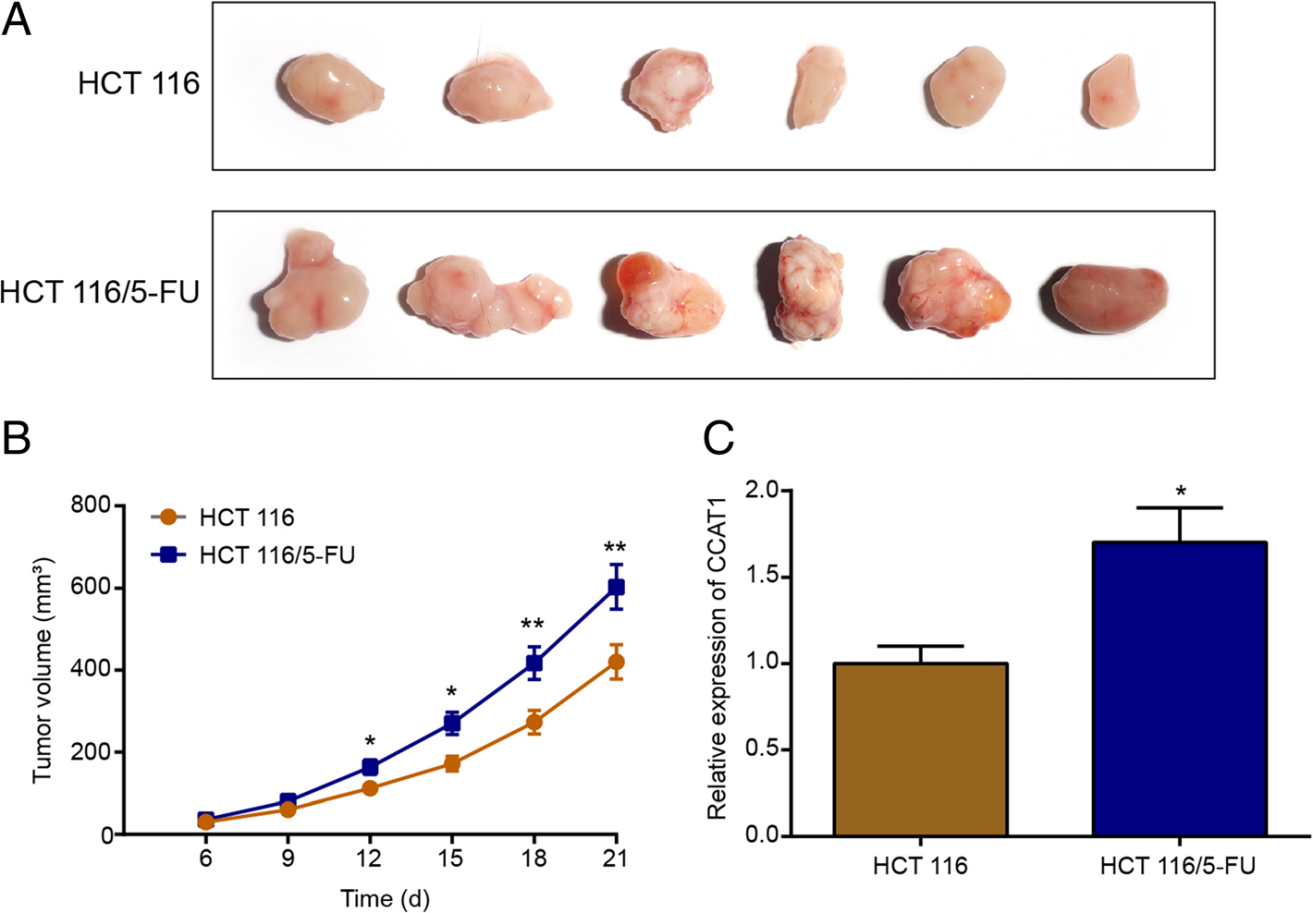
In vivo tumor growths of HCT 116 and HCT 116/5-FU cells in mice models. **a.** The formation of tumors of HCT 116 and HCT 116/5-FU cells in mice models. **b.** The in vivo growth of HCT 116 and HCT 116/5-FU xenografts were monitored by 3-day measurements on tumor volumes (*n* = 6). **c.** After 21 days, the mice were sacrificed and the tumors were removed. Tumor samples were used to detect the expression of CCAT1 by qRT-PCR. *P < 0.05, **P < 0.01, compared with HCT 116 cells in mice model

To explore the expression of CCAT1 in vivo, HCT 116 and HCT 116/5-FU cell-derived xenograft tumors were allowed to develop and grow for 3 weeks. Then, the mice were sacrificed and their tumors were excised. As shown in Fig. 6a, the tumors in the HCT 116/5-FU group grew continuously during the experimental period, whereas the tumor growth in HCT 116 mice was markedly slower compared to these values in the HCT 116/5-FU group. Figure 6b demonstrates that the tumor volume in the HCT 116/5-FU group was significantly increased compared with that in the HCT 116 group. Moreover, we detected the relative CCAT1 expression in the tumor samples after mice were sacrificed. As shown in Fig. 6c, the HCT 116/5-FU group had a higher expression of CCAT1 compared with the HCT 116 group, which confirmed the higher expression of CCAT1 in 5-FU resistant cells in vivo.

## Discussion

Chemotherapy is an important treatment for patients with advanced colonic neoplasms. 5-FU, a first-line chemotherapy for patients with colonic neoplasms, is less toxic and has a sustained inhibition on anti-tumor activity [21]. However, the generation of 5-FU resistances of colon cancer patients will inevitably lead to chemotherapy failure. Therefore, clarifying the mechanism of resistance to 5-FU in patients with colon cancer can improve the clinical efficacy [22].

Recently, multiple studies have reported that dysregulation of lncRNAs played an important role in the development of chemoresistance [23–25]. With the deepening research in colon cancer, an increasing number of lncRNAs are found involved in tumor incidence, development, invasion, migration and regulation of chemoresistance. In addition, lncRNAs may affect epigenetic status through chromatin-modifying complexes and deliver the phenotype required for multiple tumor progression and metastasis [26]. Li et al. found low expression of lncRNA SLC25A25-AS1 in colon cancer. The low expression of lncRNA SLC25A25-AS1 correlated with tumor proliferation, and lncRNA SLC25A25-AS1 could regulate chemoresistance by inhibiting the ERK / p38 signaling pathway [27]. Bian and coworkers found that lncRNA-UCA1 was significantly and highly expressed in colon cancer. The high expression of lncRNA-UCA1 promoted the proliferation of colon cancer cells and the chemotherapeutic resistance to 5-FU by inhibiting miRNA-204-5p [28].

CCAT1, an overexpressed lncRNA in colonic neoplasm tissues compared with normal tissues, promoted the proliferation and invasion of colon cancer cells. Clinically, CCAT1 is closely related to the clinical stage, lymph node metastasis and prognosis of patients [29]. Dong et al. reported that CCAT1 was significantly upregulated in nasopharyngeal carcinoma (NPC) tissues and that CCAT1 induced growth, migration and invasion and inhibited apoptosis in NPC cells [30]. Li et al. also found that CCAT1 was highly expressed in gastric cancer (GC) tissues compared with normal counterparts and that CCAT1 upregulation promoted proliferation, invasion and migration of GC cells in vitro [31]. Another study demonstrated that CCAT1 was significantly upregulated in multiple myeloma (MM) tissues and cell lines and that CCAT1 knockdown significantly inhibited cell proliferation, induced cell cycle arrest at the G0/G1 phase, promoted cell apoptosis in vitro, and suppressed tumor growth in vivo [32]. Chen et al. reported that CCAT1 inhibits cell apoptosis of renal cell carcinoma (RCC) through the upregulation of Livin protein in vitro [33]. In addition, Nissan et al. also found that CCAT1 was strongly expressed in tissues representing the early phase of tumorigenesis, in adenomatous polyps and in tumor-proximal colonic epithelium, as well as in later stages of colorectal cancer (liver metastasis, for example) [10]. Similarly, we found higher expression of CCAT1 in colon cancer, and overexpression of CCAT1 inhibited cell apoptosis of colon cancer.

Moreover, CCAT1 was found to be upregulated in DDP-resistant NSCLC cells. The expression of CCAT1 and DDP resistance, in both NSCLC cells and DDP-resistant NSCLC cells, were positively correlated [14]. Another study indicated that the expression of CCAT1 was greater in docetaxel-resistant lung adenocarcinoma cells [15]. Additionally, it was reported that the overexpression of CCAT1 resulted in significant enhancement of paclitaxel resistance in nasopharyngeal cancer cells [34]. However, there is little research about CCAT1 affecting 5-FU sensibility of human colon cancer cells. In our research, experiments demonstrated that the CCAT1 reduced the chemosensitivity of colonic neoplasm cells in vitro and decreased their apoptosis rates, which the preliminarily investigated effect of the lncRNA was the downregulation on 5-FU antitumor functions.

Accumulating evidence has indicated that lncRNAs exert its effects as competing endogenous RNA (ceRNA) [16]. In the case, lncRNAs competitively inhibit miRNAs, and miRNAs inhibit lncRNA via an Argonaute 2 complex mediated pathway [35]. In the previous studies, Lu et al. reported that the epigenetic silencing of miR-218 by CCAT1 induced an altered cell cycle transition through BMI1 and provided a new mechanism for CSE-induced lung carcinogenesis [36]. Yang et al. deduced that CCAT1 acted as a molecular sponge in regulating the biological functions of miR-143 in the FTC-133 thyroid carcinoma cell line [23]. Li et al. demonstrated that CCAT1 contributed to the growth and invasion of gastric cancer via targeting miR-219-1 [37]. Zhao et al. confirmed that miR-148a was a direct target of CCAT1 in osteosarcoma [38]. Another study by Zhang et al. reported that CCAT1 directly targeted miR-152 in intrahepatic cholangiocarcinoma (ICC) cells [19]. In the present study, we detected the expression of these microRNAs by qRT-PCR. Markedly, we found three miRNAs (miR-218, miRNA-143 and miR-152) were downregulated in 5-FU resistant cell lines and were negatively correlated with CCAT1 in colon cancer cells. Moreover, previous studies reported that miR-218 [39], miR-143 [40] and miR-152 [41] were all downregulated in colon cancer or colorectal cancer cells. A tumor suppressor miRNA, miR-218, posttranscriptionally suppressed the MACC1 expression and its metastasis-promoting abilities in colorectal cancer [39]. In addition, the lncRNA MNX1-AS1 acts as a ceRNA of miR-218-5p to facilitate the expression of SEC61A1 [42]. In colorectal cancer, miR-143 inhibited cell migration and invasion by targeting MACC1 [43]. Besides, miRNA-143 enhanced sensitivity to 5-FU in HCT 116 human colorectal cancer cells through ERPK5/NF-κB pathways [44]. By targeting DNMT1, miR-152 inhibited colorectal cancer [45]. In this study, CCAT1 may also serve as a ceRNA for miR-218, miRNA-143 and miR-152 through sponging them, and finally enhancing 5-FU chemoresistance in colon cancer cells. Despite significant progress in CCAT1 and miRNAs expression in 5-FU resistant cell lines, there are still several relevant mechanism-related questions to be solved in future studies. For example, which miRNA primarily contributes to the effects of CCAT1? Does CCAT1 promote the 5-FU resistance by regulating other miRNAs? How does CCAT1/miRNA regulate 5-FU resistance? These questions deserve in-depth investigation in future studies. In addition, the expression of γ-H2AX (a DNA damage Sensor [46]) and p53 was detected by Western Blot. The results of our study indicated that CCAT1 modulated 5-FU sensibility of human colon cancer through DNA damage, but not p53-mediated apoptosis.

c-Myc is a transcription factor that is generally recognized as an important regulator of cell cycle, proliferation, differentiation, and apoptosis [47, 48]. Chen demonstrated that c-Myc was subsequently validated as a downstream target of CCAT1 ceRNA activity and was important for CCAT1 to regulate acute myeloid leukemia (AML) progression [49]. Moreover, we also detected the protein expression of c-Myc, which was found to be positively correlated with CCAT1 in colon cancer cells. This result might explain how CCAT1 regulate 5-FU sensibility, but further research is needed.

## Conclusions

In summary, we demonstrated that the high expression of CCAT1 in colonic neoplasm cells was closely associated with the emergence, development and drug resistance of human colon cancer. The expression levels of CCAT1 in colon cancer cells were positively correlated with 5-FU resistance. siRNA-CCAT1 effectively inhibited the expression levels of CCAT1 in HCT 116/5-FU and HT-29/5-FU colon cancer cells, inhibit cell growth and enhance sensitivity of HCT 116 and HT-29 colonic neoplasm cells to 5-FU chemotherapeutic drugs. Our studies suggest that the downregulation of CCAT1 in patients could be considered as a new therapeutic approach for treating colon cancer.

## Methods

### Tissues

Sixty-seven colon cancer tissue samples were collected from the Sichuan Academy of Medical Sciences & Sichuan Provincial People’s Hospital. None of the colon cancer patients underwent radiotherapy, preoperative chemotherapy or other tumor-specific therapies. All specimens were confirmed by pathological examination, according to the WHO histological classification and grading standards. The implementation of this research was approved by the Clinical Research Ethics Committee of Sichuan Academy of Medical Sciences & Sichuan Provincial People’s Hospital. All patients read and signed informed consent before the study began, and approval was obtained from the Sichuan Academy of Medical Sciences & Sichuan Provincial People’s Hospital.

### Microarray

Total cellular RNA was extracted and cDNA was synthesized using T7 Oligo (dT) Primer as the primer. Using cDNA as a template, T7 Enzyme Mix was selected for cRNA synthesis, 100 μL of biotin was added. The salts and enzymes in cRNA were removed and purified by magnetic beads. Hybridization was performed using an Affymetrix Human Genome U133 Plus 2.0 Array chip as previously described [50]. After cRNA fragmentation (100 bp), hybridization was performed on a prehybridization chip at 45 °C for 10 min. The same volume of hybridization solution replaced the prehybridization solution. After biotinylated cRNA was labeled, hybridization was performed again, and hybridization was carried out at 45 °C for 16 h. Eluted and stained chips were scanned with an Agilent Microarray Scanner to obtain data. Data were analyzed using GeneChip Operating Software version 1.0.

### Cell lines

Human colonic neoplasm cell lines HCT 116, SW1417, HT-29, and KM12 and the human normal colonic epithelial cell line NCM460 were provided by the Chinese Academy, Medical Sciences Cancer Cell Bank (Shanghai, China) and were maintained in RPMI medium containing with 1% penicillin/streptomycin and 10% fetal bovine serum.

### Real-time quantitative reverse-transcription polymerase chain reaction (qRT-PCR)

TRIzol reagent (Invitrogen, CA, USA) was used to isolate total RNA from both cells and tissues following the instructions of the manufacturer. The Prime Script RT Reagent Kit (Takara, Japan) and SYBR Prime Script RT-PCR Kits (Takara, Japan) were used, according to the manufacturer’s protocol, to perform the reverse transcription and qRT-PCR, respectively. CCAT1 levels, which were respectively normalized to GAPDH, were calculated by the 2^-ΔΔCt^ method. One microgram of the extracted RNA was reverse-transcribed followed by cDNA preamplification. The amplified cDNA samples were loaded onto a TaqMan Low Density Array (TaqMan Human MicroRNA Array v3.0 A and B; Applied Biosystems, Foster City, CA). miRNA abundance is presented as threshold cycle (Ct) values normalized to U6 snRNA. The relative abundance of different miRNAs in the samples was expressed as fold change calculated by the comparative Ct method (2^-ΔΔCt^). miRNAs were measured using TaqMan miRNA Assay Kits (Applied Biosystems, USA) according to the manufacturer’s protocol. All analyses were performed in triplicate. CCAT1, forward primer: 5′ CATTGGGAAAGGTGCCGAGA 3′,

reverse primer: 5′ ACGCTTAGCCATACAGAGCC 3.

GAPDH, forward primer: 5′ GGGAGCCAAAAGGGTCAT 3′,

and reverse primer: 5′GAGTCCTTCCACGATACCAA 3′.

### Establishment of HCT 116 and HT-29 drug-resistant cell lines

Using continuous exposure to increasing 5-FU concentration method, HCT 116 and HT-29 cells were induced by 5-FU with a starting concentration of 0.1 μg/ml. After cells stably grew, cells were passaged for 2 to 3 generations, and the concentration of 5-FU was increased by the following concentration gradient; the induced drug concentration was 0.1 μg/ml for over 6 weeks, then exposed to 0.5 μg/ml 5-FU for over 8 weeks, and then 1 μg/ml for another 8 weeks. The drug concentration was gradually increased as described above for 30 weeks in total, and the HCT 116/5-FU and HT-29/5-FU resistant cell lines stably grew at a 5-FU concentration of no more than 2 μg/ml. Before the experiment, HCT 116/5-FU and HT-29/5-FU resistant cell lines had been cultured for 2 weeks in media without drugs until used for experiments.

### Cells transfection

The sequence of CCAT1 was synthesized and subcloned into pcDNA3.1 (Invitrogen, Shanghai, China). The siRNA to CCAT1 (si-CCAT1) was purchased from Shanghai GenePharma Co., Ltd. (Shanghai, China). The pcDNA-CCAT1 transfection was used to upregulate the expression of CCAT1 and empty pcDNA vector was used as a control. DMEM, which contained 10% fetal bovine serum, was used to retain colon cancer cells HCT 116, HT-29 HCT 116/5-FU, and HT-29/5-FU with saturated humidity and 5% CO_2_ at 37 °C in an incubator. Cells in logarithmic phase were digested and seeded (2 × 10^5^ cells per well) in 6-well cell culture plates until cells were fused 60~70%. The pcDNA3.1-CCAT1 was transfected into HCT 116 and HT-29 cell lines and siRNA-CCAT1 were transfected into HCT 116/5-FU and HT-29/5-FU cell lines for 48 h, according to Lipofectamine™ 2000 transfection reagent manual. The expression level of CCAT1 was detected by qRT-PCR.

### CCK-8 assay

Chemosensitivity was detected by CCK-8 assay. Cells were cultivated in 96-well plates treated with 5-FU (0.5 μg/mL). After 48 h, 10 μl of CCK-8 solution was added to each well and cells were stored in an incubator with saturated humidity and 5% CO_2_ at 37 °C for 4 h. Enzyme-linked immunoassay detection assay was performed at OD value at 450 nm. Each set was 6 complex wells, and statistics were the average OD value. The experiment was performed in triplicate.

### Flow cytometry analysis

Cells transfected with the indicated plasmid or negative control were harvested after 48 h. The cell density was kept at 3 × 10^5^ cells/ml. After washing with PBS, HCT 116 and HT-29 cell lines were fixed with 75% ethanol overnight after 5-FU treatment for 48 h. The next day, the cells were incubated with RNase at 37 °C for 30 min. After 5-FU treatment, propidium iodide (Sigma) was used to stain cells and flow cytometry (FACSCalibur) was used to analyze the cell cycle. Cell death was determined by staining cells with Annexin V using the ApoScan kit (Biobud, Korea) according to the manufacturer’s protocol. All samples were analyzed in triplicate.

### Western blot

Equal amounts (50 μg) of protein extracts were loaded and separated by SDS-PAGE using acrylamide gradients. The membranes were incubated for 1 h at room temperature with the indicated primary antibodies [phosphorylation of histone H2AX (γ-H2AX) (1:1000, #9718, Cell Signaling Technology (CST), Danvers, MA, USA), p53 (1:1000, #2524, CST), GAPDH (1:1000, #2118, CST)]. Horseradish peroxidase-conjugated anti-rabbit immunoglobulin IgG (1:2000, CST) was used as a secondary antibody and incubated for 1 h at room temperature. The washing procedure was repeated eight times within one hour. Immunoreactive bands were visualized by enhanced chemiluminescence (ECL; Amersham Biosciences) and exposed to Biomax L film (Kodak). For the purpose of quantification, ECL signals were digitized using LabWork software (UVP).

### In vivo tumor growth

All animal studies were designed and performed in compliance with the approved protocol by the Sichuan Academy of Medical Sciences & Sichuan Provincial People’s Hospital. Male BALB/c mice (5- to 6-week-old) were supplied by the Sichuan Academy of Medical Sciences & Sichuan Provincial People’s Hospital. The HCT 116 and HCT 116/5-FU cells (1 × 10^5^) were resuspended in PBS (50 μL) and subcutaneously injected into mice for colon tumor generation, and mice were randomly assigned to two groups (*n* = 6 mice/group), an HCT 116 group and an HCT 116/5-FU group. During the study, tumor volume in mice was monitored every 3 days. Tumors were dissected after 21 days after inoculation. The volume of tumor was calculated as follows: tumor volume = (width)^2^ × (length)/2. RNA was extracted from the dissected tumor tissue, and the content of CCAT1 was detected by qRT-PCR.

### Statistical analysis

SPSS 18.0 software and GraphPad Prism 6.0 software were used for statistical analysis, and all measurement data were expressed as the mean ± standard deviation (mean ± SD). The statistical significance of the mean values among different groups was determined using one-way ANOVA. *P* < 0.05 was considered significantly different.

## Abbreviations

5-Fu: 5-fluorouracil
AML: acute myeloid leukaemia
GC: gastric cancer
ICC: intrahepatic cholangiocarcinoma
LncRNAs: Long-chain non-coding RNAs
NC: negative control
NPC: nasopharyngeal carcinoma
SD: standard deviation
si-CCAT1: siRNA to CCAT1
